# Predictive factors of return to work after hysterectomy: a retrospective study

**DOI:** 10.1186/s12893-022-01533-y

**Published:** 2022-03-04

**Authors:** Suzanne J. Dedden, Esther V. A. Bouwsma, Peggy M. A. J. Geomini, Marlies Y. Bongers, Judith A. F. Huirne

**Affiliations:** 1 Department of Obstetrics and Gynaecology, Máxima Medisch Centrum, Veldhoven, The Netherlands; 2 GROW School of Oncology and Developmental Biology, Maastricht University, Maastricht, The Netherlands; 3 Department of Obstetrics and Gynaecology, Amsterdam UMC, Amsterdam, The Netherlands

**Keywords:** Hysterectomy, Recovery, Convalescence, Gynaecology, Surgery, Return to work, Indwelling catheter, Fast-track

## Abstract

**Purpose:**

Although hysterectomy is one of the most frequently performed gynaecological surgeries, there is a dearth of evidence on perioperative care. The aim of the current study was to identify sociodemographic, surgical-related and work-related predictors of recovery following different approaches of hysterectomy.

**Methods:**

Eligible patients for this retrospective cohort study were women who underwent vaginal, abdominal or laparoscopic hysterectomy for both benign and malignant gynaecological disease in 2014 in Máxima Medical Centre in the Netherlands. The main outcome measure was full return to work (RTW). Data were collected using a patient survey. Potential prognostic factors for time to RTW were examined in univariate Cox regression analyses. The strongest prognostic factors were combined in a multivariable model.

**Results:**

In total 83 women were included. Median time to full return to work was 8 weeks (interquartile range [IQR] 6–12). The multivariable analysis showed that higher age (hazard ratio [HR] 1.053, 95% confidence interval [CI] 1.012–1.095) and same day removal of indwelling catheter (HR 0.122, 95% CI 0.028–0.539) were predictors of shorter duration until full RTW after hysterectomy.

**Conclusions:**

This study provided insight in the predictors of recovery after hysterectomy. By identifying patient specific factors, pre-operative counselling can be individualized, changes can be made in perioperative care and effective interventions can be designed to target those factors.

## Introduction

Hysterectomy is one of the most frequently performed gynaecological surgery in women worldwide [1–3]. Nevertheless, there is still a dearth of evidence on perioperative care, which can be translated into two main gaps in current knowledge: (1) normal recovery times after hysterectomy are not well defined, and (2) important predictors of post-hysterectomy recovery have yet to be determined.

In the last two decades there are a few attempts made to describe normal trajectories after different approaches to hysterectomy, but these studies usually consist of relative small samples sizes, are characterized by a large heterogeneity in study methodology and lack standardized recovery outcome measures [4–10]. Yet, the conclusion that can be drawn from this research is that there is a wide variety of recovery times among patients undergoing similar types of surgeries, assuming there are a lot of variables that influence the length of recovery [11, 12]. However, studies designed to identify predictors of recovery have not led to uniform conclusions [13–15].

Due to these knowledge gaps, healthcare providers endeavour many challenges in providing their patients with solid, standardized, evidence-based perioperative care. Unfortunately, the lack of standardized and uniform perioperative advice leads to patients who are insecure and hold on to inappropriate recovery expectations [16, 17]. Ironically, the relation between recovery expectations and health outcomes has been established well: the duration of postoperative recovery can be significantly influenced by pre-operative counselling and education [18–20].

The aim of the current study was to identify sociodemographic, surgery-related and work-related predictors of recovery in patients following different surgical approaches of hysterectomy. Duration until full return to work (RTW) was chosen as the primary outcome, as this is a well-defined endpoint of recovery [21, 22]. The results of this study will help building the evidence base of perioperative care, by enabling the formulation of evidence-based convalescence advice after hysterectomy [23, 24]. Moreover, by identifying factors that are modifiable, changes can be made in perioperative care and effective interventions can be designed to target those factors [11]. These strategies will not only benefit patients themselves, but society as a whole, through a decrease in costs related to unnecessary prolonged recovery after hysterectomy.

## Methods and materials

### Study design

This retrospective cohort study was conducted in a tertiary teaching hospital in the Netherlands: the Máxima Medical Centre Veldhoven (Máxima MC). Ethical approval was obtained by the local Medical Ethical Committee, registered under number 15.101.

Eligible patients were women who underwent a vaginal, laparoscopic or abdominal hysterectomy for both benign and malignant indication between January 1st 2014 and January 1st 2015 and were identified through an electronically generated overview of all surgeries performed in 2014. Only patients with low grade endometrial carcinoma were included, who did not require additional adjuvant treatment. In addition, patients requiring lymph node excision, radical hysterectomy or debulking surgery did not belong to our sample either, as this type of care is centralized in the Netherland.

The preferred route for hysterectomy was the vaginal hysterectomy. If the vaginal approach was technically not possible due to insufficient mobility of the uterus or large uterine size, the laparoscopic route was preferred over the abdominal approach because it is associated with less pain, less blood loss and a rapid recovery [25].

The patients were approached and invited to participate in September 2015.

Patients received an envelope containing patient information, two informed consents, a leaflet about participating in scientific research in general. A reminder was sent after 3 and 6 weeks when no answer was received.

### Outcome measures

The main outcome of this study was sick leave duration until full RTW, defined as the number of days between the day of surgery until the actual day on which work was fully resumed.

### Potential prognostic factors

Based on a literature search of previous studies searching for factors influencing recovery after hysterectomy we identified prognostic factors for recovery [5, 16, 17].

The research team then developed a questionnaire focusing on sociodemographic, perioperative and work-related factors. Potential factors leading to a more beneficial or disadvantageous recovery were identified.

The following factors were incorporated in the questionnaire:

Sociodemographic factors:

- Age (years)
- Parity
- Nationality
- Education level
- Family composition (alone, with partner, service flat)

Perioperative factors

- Surgical approach (vaginal, laparoscopic, abdominal)
- Indication (benign or malignant)
- Uterine weight
- Operative time
- Amount of blood loss
- Presence of perioperative complication(s)
- Length of stay in hospital (number of nights)
- Time of indwelling catheter

Work-related factors

- Employment status (salaried, self-employed, housework, voluntary job)
- Physical workload (light, moderate or heavy)

All outcomes were self-reported, except from some perioperative outcomes that were obtained from medical files (indications for surgery, type of surgery, uterine weight, operative time, amount of blood loss and occurrence of complications).

### Statistical analysis

Data were analysed using Statistical Package for the Social Sciences (SPSS). Continuous variables were summarized by their mean and standard deviation (SD) when normally distributed and by their median with interquartile (IQR) otherwise. Categorical data were summarized by frequencies and percentages.

Kaplan–Meier curves were used to visualize the proportion of women returned to work over time. The log-rank test was used to compare time to RTW between different types of surgery. Potential prognostic factors for time to RTW were examined in univariate Cox regression analyses. The strongest prognostic factors were combined in a multivariable model.

## Results

**Fig. 1:**
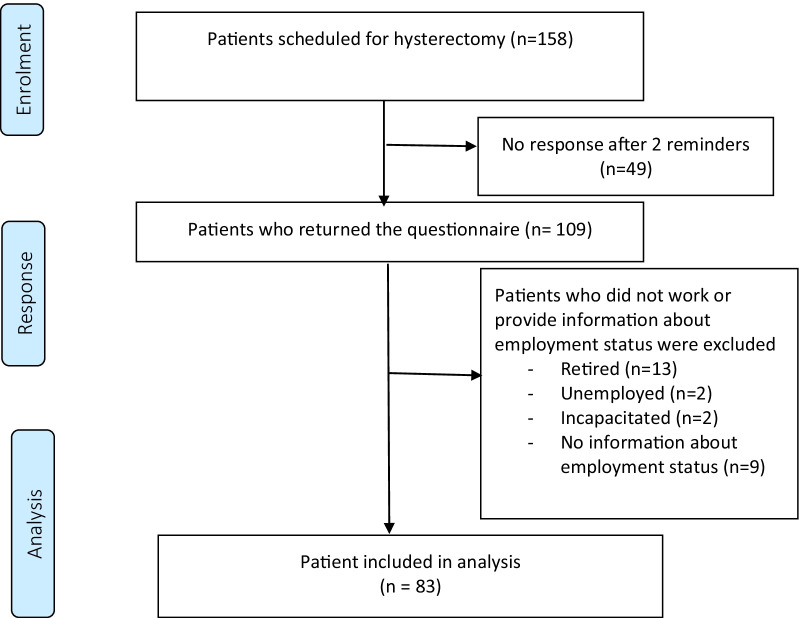
Patient flow

Between January 1 and December 31 2014, 158 women were scheduled for a hysterectomy and were approached to participate in the study. In total, 109 patients returned the questionnaire which led to a response rate of 69%. 26 patients were excluded because they did not work. The follow-up time after surgery was minimally 9 months and maximally 21 months. Figure 1 shows the patient flow in this study.

Baseline characteristics (Table 1) show that the majority of the patients had a Dutch nationality with a mean age of 48.4 years. Most patients underwent hysterectomy for benign indication. Complications after surgery were reported in 17 patients, mainly minor complications such as urinary tract infection or wound infection which were treated with antibiotics. All patients with malignancy underwent a laparoscopic hysterectomy with bilateral salphingo-oophorectomy for a low-grade endometrial carcinoma.

**Table 1:** Baseline characteristics

	Study population N = 83
*Sociodemographic factors*	
Age (years ± SD)	48.4 ± 9.9
Nationality	
Dutch	81 (98.0%)
Other	2 (2.0%)
Education level*	
Higher	27 (32.5%)
Intermediate	41 (49.4%)
Lower	15 (18.1%)
Living situation	
Alone	14 (16.9%)
With partner or family	65 (78.3%)
Other	4 (3.3%)
*Perioperative factors*	
Surgical approach	
Abdominal hysterectomy	4 (4.8%)
Laparoscopic hysterectomy	58 (69.9%)
Vaginal hysterectomy	21 (25.3%)
Indication	
Benign	75 (90.4%)
Malignant	8 (9.6%)
Complications	
None	61 (73.5%)
Perioperative	5 (6.0%)
Post-operative	17 (20.5%)
Length of stay in hospital (median nights [IQR])Time of removal of indwelling catheter	2 (1–3)
Day of surgery	14 (16.9%)
Day after surgery	63 (75.9%)
48 h or more	6 (7.0%)
*Work-related factors*	
Employment	
Salaried	54 (65.1%)
Self-employed	11 (13.2%)
Housework	16 (19.3%)
Voluntary work	2 (2.4%)
Physical workload	
Light	28 (33.7%)
Moderate	32 (38.6%)
Heavy	23 (26.5%)

Data are number of patients (%), unless otherwise indicated

*Low = preschool, primary school; intermediate = secondary school or secondary vocational education. High = higher professional education, university or postgraduate

The mean length of stay was 2 nights with an extreme outlier of 21 nights. This was a patient who had surgery because of endometrial carcinoma with multiple comorbidities and who developed a pulmonary embolism for which she was treated on the Pulmonary Department.

In most patients (75.9%) the indwelling catheter was removed the day after surgery. In November 2014 same day removal of the catheter was introduced in our clinic.

In Table 2 patient characteristics and surgery related factors are presented. Of the four patients undergoing abdominal hysterectomy laparotomic approach was only planned in one patient due to a large immobile uterus with fibroids. In two patients laparoscopic approach was converted to abdominal approach due to complications. The first patient with extensive endometriosis had an intestinal lesion with persistent haemorrhage during laparoscopy. The second patient had three previous caesareans sections and due to adhesions and extensive haemorrhage surgery was converted. One patient underwent an unplanned peripartum hysterectomy due to a placenta praevia and extreme blood loss during the Caesarean section.

**Table 2:** Peri-operative outcomes per surgical approach

Peri-operative outcomes	Surgical approach		
	Vaginal (N = 21)	Laparoscopic (N = 58)	Abdominal (N = 4)
*Patient factors*			
Age	48.5 ± 5.9	48.9 2.3 ± 2.82	41.3 ± 4.9
Parity	2.1 ± 1.1	1.6 ± 11.2	1.2 ± 1.5
*Surgery-related factors*			
Uterine weight (gr)	104.3 ± 53	312.6 ± 303	521 ± 558
Operative time (min)	68,9 ± 21	146,7 ± 63	232 ± 89
Blood loss (mL)	197.6 ± 164	119.4 ± 148	2500 ± 1853
Post-operative stay (nights)	1.6 ± 0.6	2.3 ± 2.8	7.3 ± 4.5
Complications			
None	16 (76.2%)	44 (75.9%)	1 (25%)
Perioperative	1 (4.8%)	1 (1.7%)	3 (75%)
Post-operative	4 (19%)	13 (22.4%)	0

The post-operative complications in the laparoscopic and vaginal hysterectomy group were mainly minor complications; urinary tract and superficial wound infections. One patient who underwent a laparoscopic hysterectomy had a pulmonary embolism post-operative. The patients with perioperative complication in the vaginal and laparoscopic hysterectomy both experienced more than 1000 mL of blood loss.

### Return to work

Of the working population of 83 patients, 65 (78.3%) returned to work in our follow-up.

In our clinic patient were counselled prior to surgery, regardless of surgical approach, that they should refrain from working until the check in the outpatient clinic 6 weeks after surgery.

The median time to full work resumption was 8 weeks, with a wide range of 3 to 45 weeks.

**Fig. 2:**
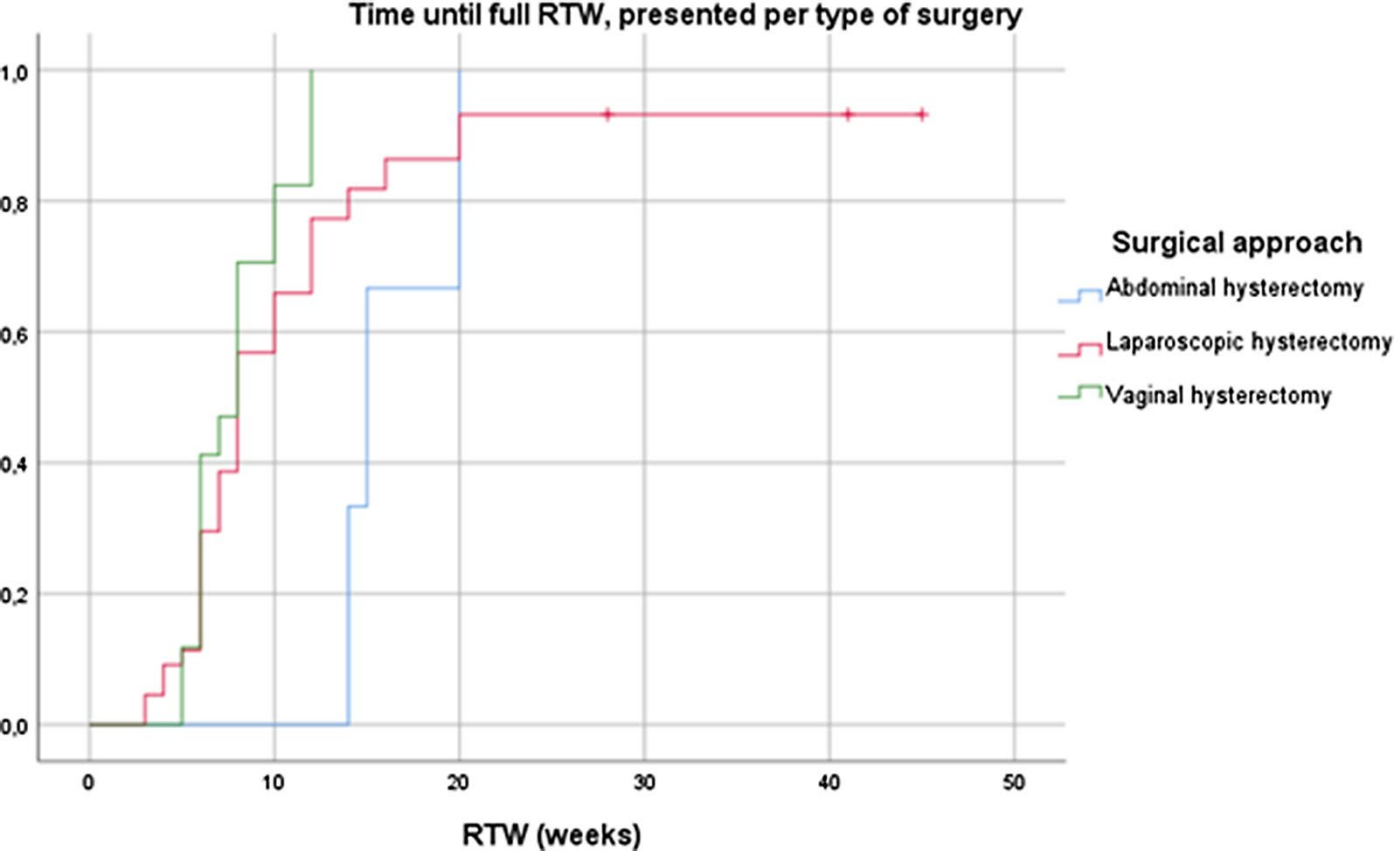
Kaplan–Meier curves for time until full RTW, presented per type of surgery

A Kaplan–Meier analysis, see Fig. 2, was used to compare time to RTW after different types of surgery. This shows a trend towards a longer period to RTW after abdominal hysterectomy than after the vaginal or laparoscopic hysterectomies. The number of patients undergoing abdominal hysterectomy is very low, the log-rank of this Kaplan Meier was not significant α 0.084.

### Univariable analysis

All women who had resumed work were included for a univariable analysis (Table 3). Age was associated with time to RTW after hysterectomy. The higher the age the shorter the time to RTW. Education level and occurrence of complications were both not related to RTW. However, duration of post-surgery stay in the hospital and duration of having an indwelling catheter was significantly related to return to work. None of the work-related factors were predictors for RTW.

**Table 3:** Univariable analysis of RTW

Predictors			Univariable analysis			
			HR	95% CI		p-value
*Patient factors*						
Age			1039	1.003–1.076		0.035
Education level						
Low vs high			1015	0.464–2.222		0.970
Medium vs high			1330	0.775–2.281		0.300
*Surgery-related factors*						
Type of hysterectomy						0.153
TLH vs TAH			0.675	0.242–1.884		0.45
TLH vs VH			1.64	0.929–2.897		0.09
Surgical complications (no)			0.998	0.53–1.89		0.99
Length of stay			0.77	0.63–0.94		0.011
Indwelling catheter						
Day of surgery vs day after surgery			0.641	0.293–1.402		0.265
Removal after 48 h vs day after surgery			0.128	0.030–0.545		0.005
*Work-related factors*						
Type of employment						0.928
Housework vs salary-employment			0.65	0.157–2.700		0.553
Housework vs self-employment			0.734	0.156–3.458		0.696
Housework vs voluntary job			0.872	0.079–0.968		0.912
Physical workload						
Moderate vs heavy			1.480	0.821–2.669		0.193
Heavy vs light			1.130	0.625–2.045		0.686

### Multivariable analysis

The three potential predictors identified by the univariate analysis (age, same day removal of the indwelling catheter and postoperative length of stay) were included in a multivariable Cox regression analysis (Table 4). In this analysis, higher age and same day removal versus after 48 h are significant.

**Table 4:** Multivariable analysis of RTW

	HR	95% CI	p-value
Age	1.053	1.012–1.095	0.011
Length of stay	0.898	0.710–1.135	0.366
Indwelling catheter			
Day of surgery vs day after surgery	0.799	0.345–1.853	0.602
Removal after 48 h vs day after surgery	0.122	0.028–0.539	0.006

These data show that higher age and same-day removal of the indwelling catheter remain predictors of shorter duration until full RTW when adjusted for age, length of post-operative stay and living situation in the multivariable analysis.

## Discussion

### Main findings

In this study we performed a retrospective cohort study in order to identify sociodemographic, surgical-related and work-related predictors of recovery following different approaches of hysterectomy. In our cohort of 83 patients, median time to full RTW was 8 weeks (IQR 6–12). The multivariable analysis showed that higher age and same day removal of indwelling catheter compared to removal after 48 h were predictors for shorter duration until full RTW after hysterectomy.

### Interpretation of the findings

The first notable finding of this study was the mean duration until full RTW of 8 weeks, which is longer than what is recommended in our clinic, which is 6 weeks for hysterectomy in general. However, these RTW-data are comparable to the findings of Vonk Noordegraaf et al. who described RTW rates after several different types of gynaecological surgery in a cohort of 148 patients in a Dutch university hospital between 2008 and 2010 [13]. In addition, it matches the growing evidence that the length of surgical recovery systematically exceeds the expected recovery times by medical specialists [7, 13, 26].

Secondly, in contrast to the aforementioned study, the level of invasiveness was not a statistically significant predictor of full RTW in our cohort. Yet, there was a trend and patients undergoing abdominal hysterectomy had a longer recovery than patients following minimal invasive approaches. The lack of finding a statistically significant relation between the level of invasiveness and the duration of sick leave may be due to the small number of patients undergoing an abdominal hysterectomy in our study.

In general, it is assumed that the impact of surgery is higher on older patients and functional recovery in the older patient takes longer than in younger patient [27, 28]. Therefore, another remarkable finding in this study was that a higher age was a significant predictor for faster recovery.

The most plausible explanation is the case mix in this study; the patients undergoing abdominal hysterectomy were younger 41 vs 48/ years old in het vaginal and laparoscopic group. These were all patients experiencing serious complications and therefore requiring longer recovery.

A possible explanation might be that younger patients have younger (more dependent) children to take care of, and therefore possibly prioritize their family tasks over work resumption, leading to prolonged duration of RTW. Physical workload may also contribute to the longer recovery. However, our study was too small to investigate these hypotheses.

### Comparison to other studies

Previous studies have shown that return to work times can be shortened with different interventions. Clayton et al. showed that pre-operative counselling about expected sickness absence duration influenced absence duration [4]. Standardized counselling about expected convalescence after uncomplicated laparoscopic cholecystectomy has shown to shorten time to return to activity and work [29].

Sanders et al. showed in a literature review on recovery after minimally invasive hysterectomy that return-to-work ranges from 3 to 12 weeks following laparoscopic hysterectomy. In an additional retrospective analysis, they showed in 31 patients that return to work was significantly faster if patients were counselled about an expected convalescence of 2 to 4 weeks compared to a more traditional counselling of 4-to-8-week recovery [30].

A study by Strozyk et al. showed in a prospective trial that prior to operation, lower preoperative psychological wellbeing and poor physical functioning led to a poorer course of recovery [15]. This is in concordance with a study by Theunissen et al. who identified ASA classification and surgery-related worries as predictors of a poorer recovery in patients undergoing hysterectomy [14]. In this study patients were counselled about an expected RTW of 6 weeks. Pre-operative counselling could be improved in our clinic based on these data, moreover it should be mentioned that convalescence advice should ideally be adapted to type of hysterectomy.

A study group in the Netherlands developed an eHealth intervention to guide women after gynaecological and abdominal surgery by providing personalized convalescence advice. Effectiveness of the intervention was well-established in three different trials demonstrating that the intervention led to a faster return to work and normal activity compared to usual care [31–33].

### Strength and weaknesses

The strength of this retrospective study is the extensive number of factors that were collected in the questionnaires post-surgery. We were therefore able to detect the role of socio-demographic factors, perioperative and work-related factors. Also, there was a considerable response-rate among the participants of 69%.

We included patients undergoing laparoscopic, abdominal and vaginal hysterectomy in this study to investigate all women undergoing hysterectomy. However, this introduced a heterogeneity in this population. In previous trials it is established that abdominal hysterectomy is associated with a longer recovery, probably due to the higher degree of invasiveness [34]. Three of the four patients that underwent an abdominal hysterectomy in our study, experienced major complications.

Our study also has limitations. To start, we potentially failed to collect data on some essential prognostic factors, such as comorbidity, pre-operative mobility and type of occupation. However, the prognostic factors we included were retrieved from a thorough literature search. Physical workload was used as a proxy for occupation.

Another limitation is the retrospective data collection, therefore recall bias could be present. In this study we focussed on return to work because this is a good-defined endpoint, however patients were counselled prior to surgery that they should refrain from working until the check in the outpatient clinic 6 weeks after surgery. The fact that all women received the same advice, could explain why the level of invasiveness turned out not to be a predictor of duration until return to work.

During the end of the study period, we started removing the indwelling catheter on the day of surgery. This might have led to a shift in attitude towards fast recovery, however this attitude was not measured in our study.

### Clinical implications

The important finding of this study is the observation that immediate removal of indwelling catheter is a significant predictor of recovery after surgery. In previous studies immediate catheter removal had shown to be safe and feasible [35]. Therefore immediate catheter removal was introduced in November 2014 in our clinic after uncomplicated laparoscopic hysterectomy [36] This is an example of a modifiable factor that can lead to significant improved outcomes once it has found its way into routine surgical care.

By identifying predictors of recovery, successful strategies to enhance recovery can be designed. Faster recovery is important, not only from the perspective of the patients, as it also reduces societal costs associated with lost productivity following surgery. This study underlines the importance of same day removal of an indwelling catheter if applicable, not only to reduce duration of hospitalization but even to enhance long term recovery as measured with RTW.

## Conclusions

In the current study it was demonstrated that fast removal of a catheter after a hysterectomy is a predictor of fast recovery. Moreover, in contrast to general belief, an older age does not necessarily lead to slower recovery. Identifying predictors of recovery in different populations should be an important future field of research as it will contribute to evidence based perioperative care.

## Data Availability

The dataset used and analysed during the current study are available from the corresponding author on reasonable request.

## Abbreviations

RTW: Full return to work
SPSS: Statistical Package for the Social Sciences
SD: Standard deviation
IQR: Interquartile range
TLH: Total laparoscopic hysterectomy
TAH: Total abdominal hysterectomy
VH: Vaginal hysterectomy
